# Comparative Functional Traits of Bamboo Monospecific Stands and Bamboo–Casuarina Mixed Stands in Coastal Sandy Land Under Different Silvicultural Regimes

**DOI:** 10.3390/plants15162487

**Published:** 2026-08-16

**Authors:** Yinghui Zhang, Hang Tao, Guiping Wan, Lulu Pu, Tianyou He, Lingyan Chen, Liguang Chen, Jundong Rong, Yushan Zheng

**Affiliations:** 1 Fujian Vocational College of Agriculture, Fuzhou 350007, China; zhyh61@126.com; 2 College of Forestry, Fujian Agriculture and Forestry University, Fuzhou 350002, China; hangtaoc@163.com (H.T.);; 3 College of Landscape Architecture and Art, Fujian Agriculture and Forestry University, Fuzhou 350002, China

**Keywords:** silvicultural regimes, coastal sandy land, bamboo monospecific stands, bamboo-Casuarina mixed stands, functional traits

## Abstract

Coastal sandy ecosystems face compounded abiotic stresses, including nutrient-poor soils, strong winds, and salt spray, making vegetation restoration highly challenging. Plant functional traits are key indicators of adaptive strategies under environmental stress, yet systematic studies on leaf, twig, and root functional traits of bamboo stands under different silvicultural regimes in coastal sandy land remain scarce. This study examined six stand types on Dongshan Island, Fujian Province: monospecific stands of *Bambusa oldhamii* Munro, *Phyllostachys nidularia f. farcata* Wen, and *Bambusa tuldoides* ‘Swolleninternode’, and their corresponding mixed stands with *Casuarina equisetifolia* L. (bamboo-to-*C. equisetifolia* ratio 7:3). Eighteen morphological and structural indices spanning three organ categories, leaf morphology (leaf area, specific leaf area [SLA], leaf tissue density [LTD], etc.), twig structure (wood density, dry matter content, etc.), and root morphology (specific root length [SRL], specific root surface area [SRA], root tissue density [RTD], etc.), were measured and analysed using Pearson correlation and principal component analysis (PCA). Results: (1) Coefficients of variation (CV) for leaf and twig traits ranged from 11.29% to 74.61%; leaf area (CV = 74.61%) and twig wood density (CV = 71.91%) were most variable, indicating high phenotypic plasticity of bamboo in coastal sandy environments. (2) Twig wood density in monospecific stands of *B. oldhamii* (0.346 g cm^−3^) was significantly higher than in all other stands (*p* < 0.05), reflecting a conservative water-transport strategy; mixed stands of *B. oldhamii* had significantly higher SRL and SRA than other stands (*p* < 0.05), indicating stronger root resource-acquisition capacity. (3) PCA revealed that leaf area, leaf volume, SLA, LTD, SRL, average root diameter, total root volume, total root surface area, and twig wood density (TWD) were the key traits distinguishing stands under different silvicultural regimes; monospecific stands scored higher overall than mixed stands, reflecting superior leaf, twig, and root functional coordination. Different silvicultural regimes significantly shape the functional adaptive strategies of bamboo in coastal sandy land. Bamboo plants integrate leaf, twig, and root traits in a coordinated, resource-conservative manner to withstand coastal stresses. These findings provide a theoretical basis for bamboo species selection and mixed-stand configuration in coastal shelterbelt management.

## 1. Introduction

Plant functional traits are key indicators of plant adaptive strategies under varying environmental conditions, reflecting physiological and ecological mechanisms within ecosystems [1,2]. Due to uneven distribution of water, nutrients, and light in natural environments, plants regulate functional traits to withstand environmental stress and sustain growth and reproduction [3]. Plants have evolved coordinated morphological adjustments, including changes in leaf area, dry matter content and thickness [4], branching patterns [5], and root morphology and architecture [6], to optimise resource acquisition under heterogeneous conditions. Coastal sandy soils are characterised by low fertility, poor water retention, and elevated salinity, making vegetation restoration highly dependent on the phenotypic plasticity of plant functional traits. Investigating how different silvicultural regimes shape these traits is therefore critical for promoting sustainable management and enhanced ecological function of coastal shelterbelts.

Among coastal protection species, monospecific stands of *Casuarina equisetifolia* L. have been extensively studied for their roles in sand fixation, windbreak, biomass accumulation, and nutrient cycling [7]. However, long-term monoculture management has led to soil nutrient depletion, microbial imbalance, and reduced ecological function [8,9], prompting exploration of mixed-stand configurations. Research shows that mixed stands improve soil bulk density and porosity, collectively enhancing protective efficacy [10]. Nevertheless, existing studies largely focus on *C. equisetifolia* itself, leaving the functional trait responses of interplanted companion species, particularly bamboo, to different silvicultural regimes poorly understood.

With growing interest in bamboo driven by China’s carbon-neutrality goals and the bamboo-for-plastics strategy, bamboo (subfamily Bambusoideae, family Poaceae) has attracted increasing research attention. Bamboo grows rapidly, produces high yields, and, owing to its unique clonal root system, performs crucial ecological functions including soil stabilisation, erosion control, and improvement of sandy-soil fertility [11,12]. Coastal bamboo research has addressed windbreak and sand-fixation performance, soil physicochemical improvement, and ecosystem restoration [13,14,15,16]. However, most studies have measured individual traits in isolation; systematic investigations of the multi-organ (leaf, twig, root) functional trait coordination and its underlying adaptive mechanisms under contrasting silvicultural regimes remain scarce [17,18,19]. In particular, it is unclear whether bamboo species adjust their resource-acquisition and resource-conservation traits synergistically when grown in monospecific versus mixed-stand configurations.

To address this gap, this study proposes the following hypothesis: mixed-stand cultivation will shift bamboo resource-acquisition strategies, producing coordinated changes in leaf, twig, and root functional traits; specifically, resource-acquisition traits (e.g., SLA, SRL) will be higher in mixed stands, while resource-conservation traits (e.g., LTD, RTD) will be lower compared with monospecific stands. Bamboo’s clonal growth habit, which enables resource sharing among ramets via underground rhizomes [20,21], may impose genetic constraints on trait plasticity, making stand-level differences more subtle than in non-clonal species. To test this hypothesis, this study established six stand types on Dongshan Island, Fujian Province, comprising monospecific stands of three bamboo species, *Bambusa oldhamii* (A), *Phyllostachys nidularia f. farcata* (B), and *Bambusa tuldoides* ‘Swolleninternode’ (C), and their corresponding mixed stands with *C. equisetifolia* (D, E, F). By quantifying 18 functional trait indices across leaves, twigs, and roots, this study aims to (i) characterise how silvicultural regimes affect bamboo functional traits, (ii) reveal the phenotypic plasticity and coordinated adaptive strategies of bamboo in coastal sandy environments, and (iii) provide a theoretical basis for species selection and sustainable management of coastal shelterbelts.

## 2. Materials and Methods

### 2.1. Study Area

**Figure 1:**
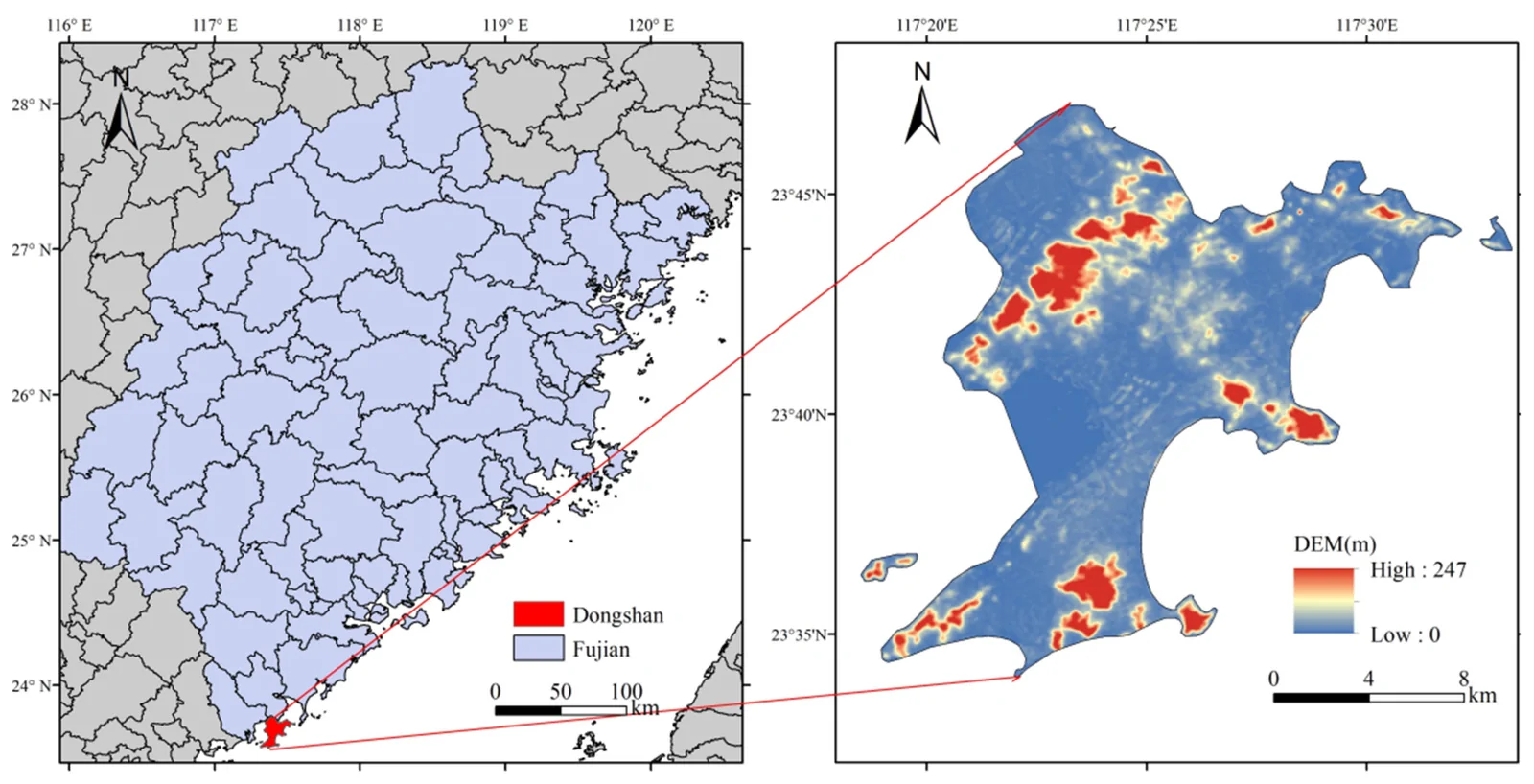
Location of the study sites on Dongshan Island, Fujian, China.

The experimental site is located on Dongshan Island, Dongshan County, Zhangzhou City, Fujian Province (23°40′ N, 118°18′ E), in a typical subtropical monsoon climate zone (Figure 1). The region has distinct wet (May–September) and dry (November–February) seasons. Annual mean temperature is 20.8 °C (max. 36.6 °C, min. 3.8 °C); mean annual precipitation is 1113.9 mm, and evaporation reaches 2013.2 mm. The island experiences strong winds (≥6 m s^−1^) on 98 days yr^−1^, mostly from October to March, with a mean annual wind speed of 7.1 m s^−1^. Typhoons primarily occur in July–August. Soil is predominantly coastal sandy loam, with loose texture and poor water and nutrient retention [22]. Bamboo plots were situated on gently sloping sandy terrain (gradient 2–10°, elevation 5–10 m, 300–600 m from the eastern coastline).

### 2.2. Experimental Materials and Plot Design

Three bamboo species were selected: *Bambusa oldhamii* (A/D), *Phyllostachys nidularia f. farcata* (B/E), and *Bambusa tuldoides* ‘Swolleninternode’ (C/F). Two silvicultural regimes were employed: monospecific stands (A–C) and mixed stands interplanted with *Casuarina equisetifolia* at a bamboo-to-*C. equisetifolia* ratio of 7:3 (D–F). Three replicate 20 m × 20 m plots were established per stand type (18 plots total). All bamboo species had been established for ≥5 yr without artificial management since introduction. Individual culm measurements were conducted in each plot to determine basic stand characteristics (Table 1).

### 2.3. Measurement Indicators and Methods

#### 2.3.1. Leaf Morphological Indicators

Within each plot, three well-developed standard bamboo plants were selected for trait measurement. From each plant, 8–10 branches were collected from the upper, middle, and lower canopy layers, and mature, undamaged leaves were sampled. Fresh leaf mass was measured with an electronic balance; leaf thickness with a thickness gauge; leaf area was scanned with an Epson Perfection V700 Photo scanner (Seiko Epson Corporation, Suwa, Nagano, Japan) and quantified in ImageJ 1.53k (National Institutes of Health, Bethesda, MD, USA; https://imagej.net/ij/). Leaves were immersed in deionised water for 12 h in the dark to obtain saturated fresh mass, then oven-killed at 105 °C for 30 min and dried at 80 °C for 72 h to constant mass. Indices were computed as follows:

Leaf dry matter content (LDMC) = Dry leaf mass/Leaf saturated fresh mass(1)

Specific leaf area (SLA) = Leaf area/Dry leaf mass(2)

Leaf volume (LV) = Leaf area × Leaf thickness(3)

Leaf tissue density (LTD) = Dry leaf mass/Leaf volume(4)

#### 2.3.2. Twig Morphological Indicators

Branches from all four cardinal directions were collected from each standard plant (3–5 per direction), labelled, and returned to the laboratory. After removing leaves and attachments, twig length and diameter were measured with a digital calliper. Twigs were soaked overnight, surface-dried, and saturated fresh mass recorded (precision 0.01 g); volume was determined by water displacement. Twigs were then oven-dried at 80 °C for 72 h to constant mass. Twig wood density (TWD) and dry matter content (TDMC) were calculated.

One-way ANOVA revealed significant differences for twig length (F(5,42) = 5.43, *p* = 0.0006, η^2^p = 0.392) and twig diameter (F(5,42) = 3.68, *p* = 0.0075, η^2^p = 0.305). Two-way ANOVA showed significant species × regime interaction for twig diameter (*p* = 0.003, η^2^p = 0.240, Supplementary Table S1).

Twig wood density (TWD) = Twig dry mass/Twig volume(5)

Twig dry matter content (TDMC) = Twig dry mass/Twig saturated fresh mass(6)

#### 2.3.3. Root Morphological Indicators

Three 1 m × 1 m subplots were established in each stand type. After felling standing bamboo at ground level, root systems were excavated with minimal breakage, brushed clean of adhering soil, and transported to the laboratory. Only fine roots (diameter ≤ 2 mm) were included in morphological analyses. After excavation, coarse roots (diameter > 2 mm) and rhizomes (severed at plot boundaries) were removed. After washing with low-temperature deionised water and air-drying the surface, roots were scanned with a root scanner and analysed in WinRHIZO Pro 2019a (Regent Instruments Inc., Québec City, QC, Canada) for total root length, total root surface area, average root diameter, and total root volume. Derived indices were calculated; root order separation was not performed; all fine roots from each subplot were pooled for analysis.

One-way ANOVA revealed highly significant differences among stand types for total root length (F(5,12) = 18.02, *p* < 0.001, η^2^p = 0.882), average root diameter (F(5,12) = 63.74, *p* < 0.001, η^2^p = 0.964), and specific root surface area (F(5,12) = 181.75, *p* < 0.001, η^2^p = 0.987). Two-way ANOVA (Supplementary Table S1) revealed exceptionally strong species × regime interactions for specific root surface area (*p* < 0.001, η^2^p = 0.985) and average root diameter (*p* < 0.001, η^2^p = 0.939), indicating highly species-dependent responses to silvicultural regime for root morphology.

Specific root length (SRL) = Root length/Root dry mass(7)

Specific root surface area (SRA) = Root surface area/Root dry mass(8)

Root tissue density (RTD) = Root dry mass/Root volume(9)

### 2.4. Data Processing and Analysis

Data were log(x + 1)-transformed to improve normality and reduce heteroscedasticity. Prior to analysis of variance (ANOVA), normality of residuals was assessed by Shapiro–Wilk tests and homogeneity of variance by Levene’s test. One-way ANOVA compared trait means among the six stand types, followed by Tukey HSD post hoc tests (α = 0.05) for pairwise comparisons. Results are reported as F(df_between_, df_within_) = F-value, *p*-value, with partial eta-squared (η^2^p) as a measure of effect size. Most traits met assumptions of normality and variance homogeneity after log transformation; traits with large sample sizes (*n* ≥ 90 for leaf traits, *n* = 48 for twig traits, *n* = 18 for root traits) showed acceptable robustness to minor departures from normality given the central limit theorem.

To directly test the effects of bamboo species (3 levels: *B. oldhamii*, *P. nidularia f. farcata*, *B. tuldoides*), silvicultural regime (2 levels: monospecific, mixed), and their interaction, this study performed two-way ANOVA on all functional traits (Supplementary Table S1). Species had significant main effects on most traits (*p* < 0.05); silvicultural regime significantly affected leaf thickness, leaf area, SLA, and leaf volume (*p* < 0.01); significant species × regime interactions were detected for leaf area (*p* < 0.001, η^2^p = 0.473), leaf volume (*p* < 0.001, η^2^p = 0.320), SLA (*p* = 0.036, η^2^p = 0.076), leaf dry matter content (*p* = 0.013, η^2^p = 0.253), twig diameter (*p* = 0.003, η^2^p = 0.240), twig wood density (*p* = 0.013, η^2^p = 0.187), and multiple root traits including average root diameter (*p* < 0.001, η^2^p = 0.939) and specific root surface area (*p* < 0.001, η^2^p = 0.985), indicating species-dependent responses to silvicultural regime.

Principal component analysis (PCA) was conducted in SPSS Statistics 21.0 (IBM Corp., Armonk, NY, USA). All 18 functional trait indices were Z-score standardised prior to analysis to eliminate unit effects. Principal components (PCs) with eigenvalues > 1 were retained, subject to a cumulative variance threshold of ≥85%. Traits with absolute factor loadings >0.75 on any retained PC were designated key response traits. Pearson correlation analysis assessed trait relationships; figures were produced in Origin 2020 (OriginLab Corporation, Northampton, MA, USA).

Assumption tests: For leaf traits, Shapiro–Wilk tests confirmed normality (*p* > 0.05) for LDMC, LA, and SLA after log transformation; Levene’s test showed acceptable homogeneity for LDMC (*p* = 0.73). For twig traits, all four morphological traits (TL, TD, TV, TWD) met both normality (Shapiro *p* > 0.19) and homogeneity (Levene *p* > 0.07) assumptions. For root traits reconstructed from summary statistics, all seven traits satisfied both assumptions (Shapiro *p* > 0.12, Levene *p* > 0.59). Traits with minor departures from normality but large sample sizes (*n* ≥ 90) were retained given robustness to violations under the central limit theorem.

Principal component analysis (PCA) was conducted in SPSS 21.0. All 18 functional trait indices were Z-score standardised prior to analysis to eliminate unit effects. Principal components (PCs) with eigenvalues > 1 were retained, subject to a cumulative variance threshold of ≥85%. Traits with absolute factor loadings > 0.75 on any retained PC were designated key response traits (a loading of 0.75 implies that the variable accounts for ≥56% of the variance of that PC, indicating strong representativeness). A comprehensive score for each stand was computed as F = Σ(contribution rate_i_ × PC_i_ score)/cumulative contribution rate, weighting each PC by its proportion of explained variance, and used to rank stands by overall functional trait performance.

## 3. Results and Analysis

### 3.1. Differences in Leaf Functional Traits Among Stand Types

**Figure 2:**
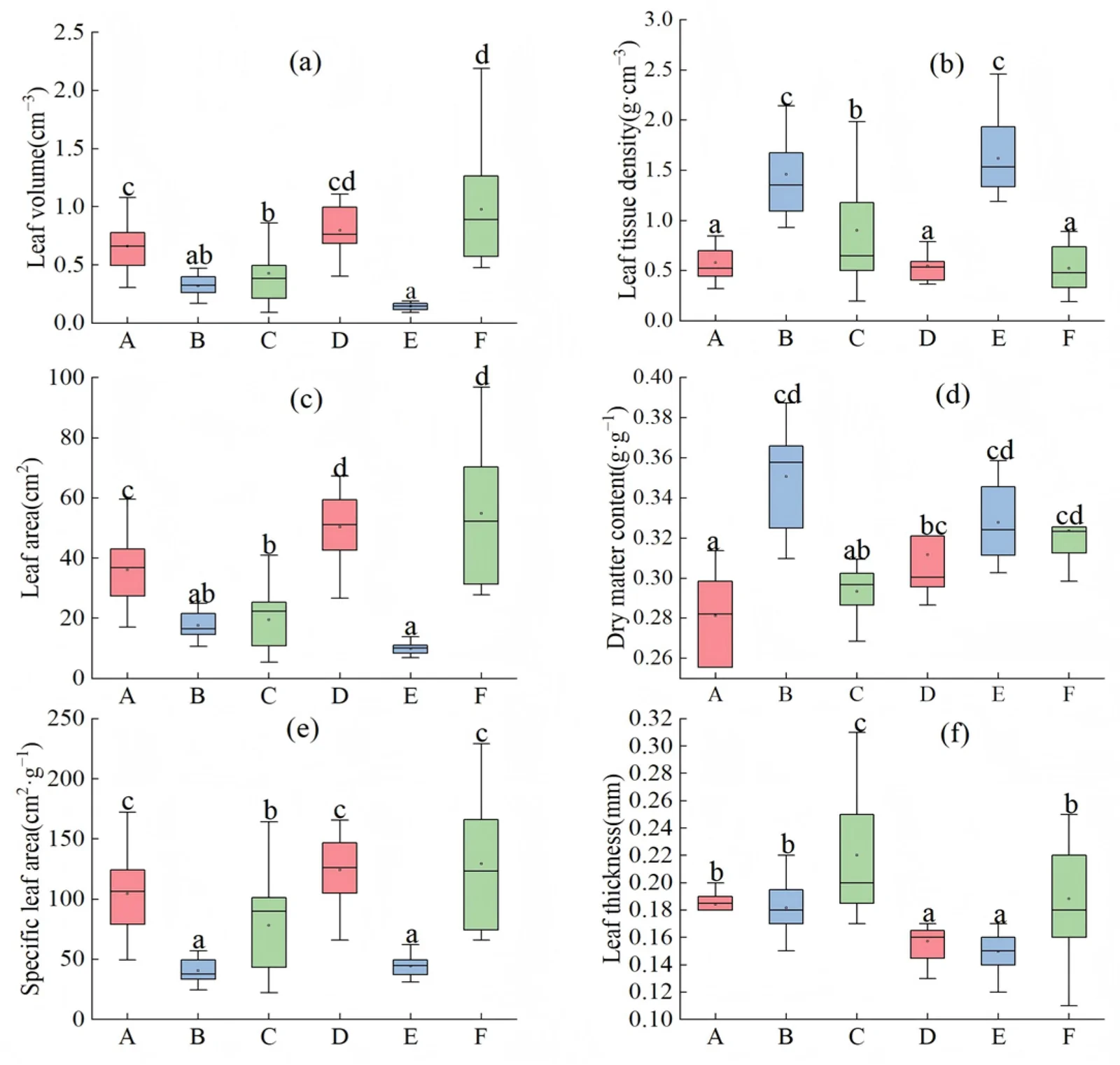
Differences in leaf functional traits among different bamboo stand types. *n* = 3 plots per stand type. (**a**) Leaf volume; (**b**) leaf tissue density (LTD); (**c**) leaf area; (**d**) leaf dry matter content (LDMC); (**e**) specific leaf area (SLA); (**f**) leaf thickness. Stand codes on the *x*-axis: A, monospecific stands of *B. oldhamii*; B, monospecific stands of *P. nidularia f. farcata*; C, monospecific stands of *B. tuldoides* ‘Swolleninternode’; D, mixed stands of *B. oldhamii* × *C. equisetifolia*; E, mixed stands of *P. nidularia f. farcata* × *C. equisetifolia*; F, mixed stands of *B. tuldoides* ‘Swolleninternode’ × *C. equisetifolia*. Different lowercase letters (a, b, c, d) above the boxes indicate significant differences among stands following Tukey HSD post hoc tests (*p* < 0.05); shared letters (e.g., ab, bc, cd) indicate no significant difference (*p* > 0.05). Boxes show the median and interquartile range, whiskers the range, and dots the mean.

*Phyllostachys nidularia f. farcata* exhibited greater LDMC and LTD than the other bamboo species, whereas its leaf area, SLA, and leaf volume were comparatively smaller (Figure 2). Leaf thickness in all three monospecific stands was significantly greater than in corresponding mixed stands (*p* < 0.05). *B. oldhamii* and *B. tuldoides* ‘Swolleninternode’ showed similar trends: leaf area was significantly higher in mixed stands than in monospecific stands (*p* < 0.05). SLA and LDMC of *B. tuldoides* ‘Swolleninternode’ were significantly higher in mixed stands (*p* < 0.05), whereas no significant differences were detected for *P. nidularia f. farcata* or *B. oldhamii* across silvicultural regimes (*p* > 0.05). The same trend extended to leaf volume and LTD, indicating that silvicultural regime had the greatest effect on *B. tuldoides* ‘Swolleninternode’.

One-way ANOVA revealed significant differences among stand types for most leaf traits: LT (F(5108) = 13.61, *p* = 0.0000, η^2^p = 0.387); LA (F(5,84) = 49.22, *p* = 0.0000, η^2^p = 0.746); SLA (F(5,84) = 27.31, *p* = 0.0000, η^2^p = 0.619). Two-way ANOVA (Supplementary Table S1) showed significant species × regime interaction for leaf area (*p* < 0.001, η^2^p = 0.473), indicating species-dependent responses to silvicultural regime.

### 3.2. Differences in Twig Functional Traits Among Stand Types

**Figure 3:**
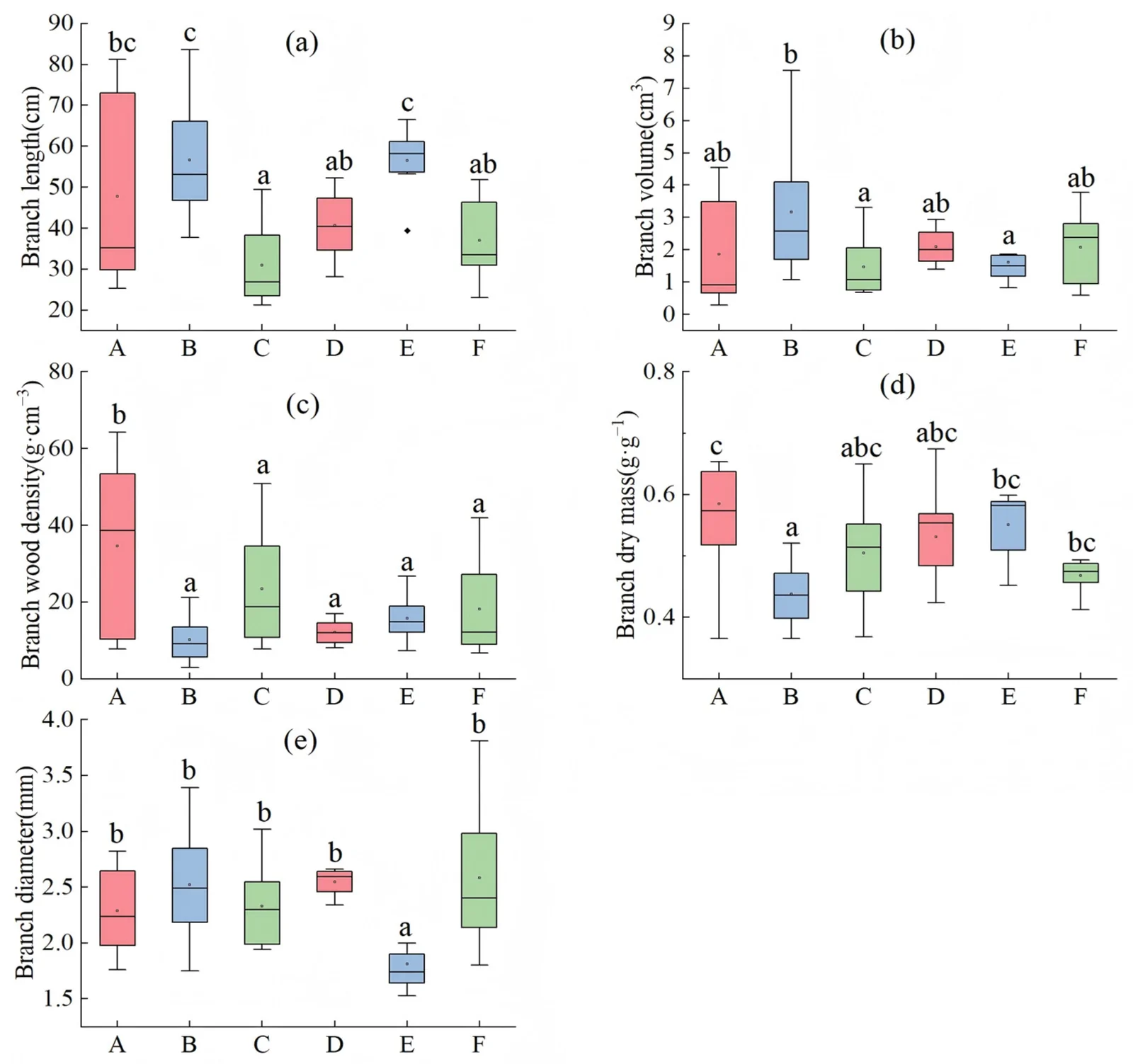
Differences in twig functional traits among different bamboo stand types. *n* = 3 plots per stand type. (**a**) Twig length; (**b**) twig volume; (**c**) twig dry mass density (twig wood density, TWD); (**d**) twig dry matter content (TDMC); (**e**) twig diameter. Stand codes (A–F) as in Figure 2. Different lowercase letters (a, b, c) above the boxes indicate significant differences among stands following Tukey HSD post hoc tests (*p* < 0.05); shared letters (e.g., ab, abc, bc) indicate no significant difference (*p* > 0.05).

Twig length and volume in monospecific stands of *B. tuldoides* ‘Swolleninternode’ (C) were significantly lower than in other stands (Figure 3). Twig diameter and volume in monospecific stands of *P. nidularia f. farcata* (B) were significantly greater than in mixed stands (E) (*p* < 0.05); no significant differences were found between monospecific and mixed stands of *B. oldhamii* or *B. tuldoides* ‘Swolleninternode’ (*p* > 0.05). Twig wood density (TWD) in monospecific stands of *B. oldhamii* (0.346 g cm^−3^) was significantly higher than in all other stands (*p* < 0.05); no significant differences existed among the remaining five stands. TDMC in mixed stands of *P. nidularia f. farcata* significantly exceeded monospecific stands, whereas the opposite held for *B. oldhamii* (monospecific > mixed; *p* < 0.05).

### 3.3. Differences in Root Morphological Traits Among Stand Types

Root morphology varied substantially among bamboo stand types (Table 2). Total root length was highest in monospecific stands of *B. oldhamii* (45.6 m m^−2^), significantly exceeding mixed stands (D) (*p* < 0.05). Total root surface area ranked as: monospecific *P. nidularia f. farcata* (B) > monospecific *B. oldhamii* (A) > mixed *B. oldhamii* (D) > monospecific *B. tuldoides* ‘Swolleninternode’ (C) > mixed *B. tuldoides* ‘Swolleninternode’ (F) > mixed *P. nidularia f. farcata* (E); all pairwise differences were significant (*p* < 0.05). Mixed stands of *P. nidularia f. farcata* (E) had significantly lower total root length, root surface area, total root volume, and SRL than other stands (*p* < 0.05). Average root diameter was highest in monospecific stands of *P. nidularia f. farcata* (B; 1.69 mm) and lowest in mixed stands (E; 0.96 mm). Mixed stands of *B. oldhamii* (D) had significantly higher SRL and SRA than all other stands (*p* < 0.05) but the lowest RTD (0.29 g cm^−3^); mixed stands of *P. nidularia f. farcata* (E) had the highest RTD (1.72 g cm^−3^).

### 3.4. Variation in Leaf and Twig Functional Traits

Coefficients of variation (CV) for leaf and twig functional traits ranged from 11.29% to 74.61% across stand types (Table 3). Mean CVs were 48.12% for leaf traits and 42.80% for twig traits, indicating high variability in both organ categories. Leaf area showed the greatest variability (CV = 74.61%), followed by twig wood density (CV = 71.91%); both represent high-plasticity traits sensitive to environmental stress. The lowest CV was observed for LDMC (11.29%), with twig diameter and TDMC also showing moderate variation (CV = 10–25%), reflecting stronger genetic constraints on these biomechanically and physiologically fundamental traits.

### 3.5. Correlations Among Leaf, Twig, and Root Functional Traits

**Figure 4:**
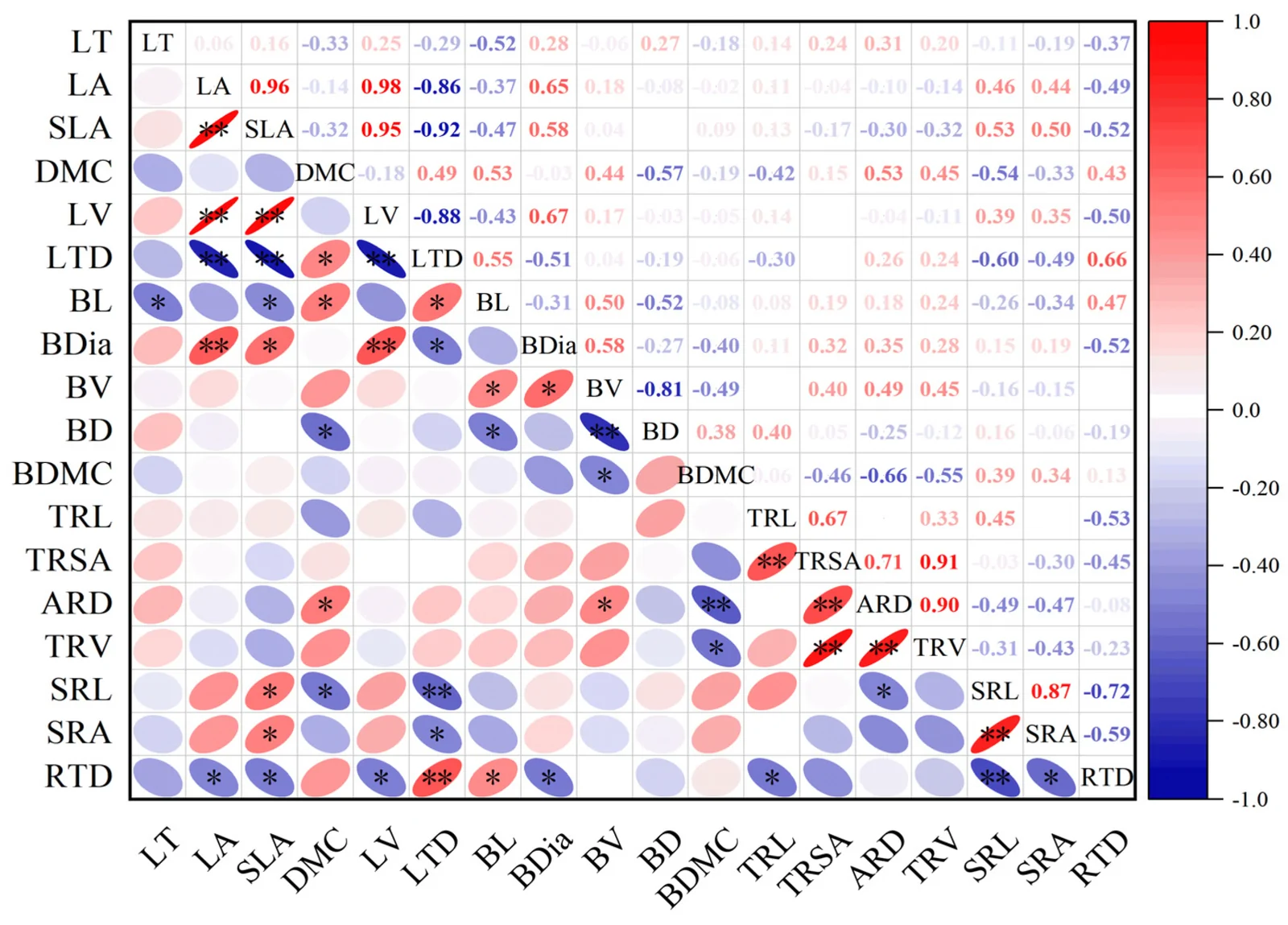
Pearson correlation matrix of leaf, twig, and root functional traits. Significant correlations (*p* < 0.05) are highlighted. *n* = 3 plots per stand type. Coefficients are given in the upper triangle and the corresponding ellipses in the lower triangle, where ellipse colour and eccentricity scale with the sign and magnitude of the correlation. * *p* < 0.05; ** *p* < 0.01. Abbreviations: LT = leaf thickness, LA = leaf area, SLA = specific leaf area, DMC = leaf dry matter content, LV = leaf volume, LTD = leaf tissue density, BL = twig length, BDia = twig diameter, BV = twig volume, BD = twig dry mass density (twig wood density, TWD), BDMC = twig dry matter content (TDMC), TRL = total root length, TRSA = total root surface area, ARD = average root diameter, TRV = total root volume, SRL = specific root length, SRA = specific root surface area, RTD = root tissue density.

Pearson correlation analysis identified 27 trait pairs with significant correlations (*p* < 0.05) and 18 with highly significant correlations (*p* < 0.01), indicating broad coordination across leaf, twig, and root functional traits (Figure 4). Correlations were stronger within organ types, but several significant inter-organ associations were detected. Notably, twig diameter was strongly positively correlated with leaf area and leaf volume (*p* < 0.01); twig TDMC was strongly negatively correlated with average root diameter (*p* < 0.01); and LTD was strongly negatively correlated with SRL and strongly positively correlated with RTD (both *p* < 0.01), revealing structural and functional coordination between leaf and root organs.

### 3.6. Key Traits Distinguishing Silvicultural Regimes Identified by Principal Component Analysis

Z-score standardisation followed by PCA yielded five principal components (PCs) with eigenvalues > 1 (eigenvalues: 6.206, 4.390, 2.631, 1.794, 1.015; cumulative variance = 89.084% > 85% threshold), comprehensively retaining original data information (Table 4). Traits with absolute loadings > 0.75 on any PC were selected as key response traits (this threshold implies ≥ 56% of the PC variance is explained by that trait, indicating strong representativeness). By this criterion, nine traits were identified as key: leaf area, leaf volume, SLA (PC1; resource acquisition), LTD (PC1; resource conservation), SRL (PC1; root acquisition capacity), average root diameter, total root volume, and total root surface area (PC2; root structural capacity), and twig wood density (PC3; hydraulic safety). Together, these traits represent the core functional dimensions—leaf carbon capture, leaf resource conservation, and root water–nutrient absorption—through which bamboo stands respond to silvicultural regime.

The comprehensive score ranking (Table 5) showed that monospecific stands of *P. nidularia f. farcata* (B) and *B. oldhamii* (A) ranked first and second, respectively. Ecologically, this ranking reflects differences in the degree of leaf–twig–root functional coordination under contrasting silvicultural regimes: monospecific stands, free from interspecific competition, sustain more stable multi-organ functional trait combinations and thus achieve higher overall resource-use coordination scores. In mixed stands, interspecific competition from *C. equisetifolia* may divert carbon investment across organs, weakening leaf–root coordination and reducing composite scores. Importantly, the composite score represents functional coordination efficiency rather than overall ecological service value; mixed stands may retain distinct advantages in soil improvement and biodiversity support [23] over longer timescales.

Complete two-way ANOVA results for all 18 functional traits, including F-statistics, degrees of freedom, *p*-values, and partial eta-squared effect sizes for species main effects, regime main effects, and species × regime interactions, are presented in Supplementary Table S1.

## 4. Discussion

### 4.1. Variation Patterns of Leaf, Twig, and Root Traits Under Different Silvicultural Regimes

The primary hypothesis—that mixed-stand cultivation would increase resource-acquisition traits (SLA, SRL) and decrease resource-conservation traits (LTD, RTD) relative to monospecific stands—received partial support. Two-way ANOVA revealed significant silvicultural regime main effects on leaf thickness (*p* < 0.001), leaf area (*p* < 0.001), SLA (*p* < 0.001), and leaf volume (*p* < 0.01), with mixed stands generally showing higher values (Supplementary Table S1). However, significant species × regime interactions for leaf area (*p* < 0.001), leaf volume (*p* < 0.001), and SLA (*p* = 0.036) indicated that regime effects were species-dependent rather than universal. For *B. oldhamii*, mixed stands exhibited significantly higher SRL and SRA, consistent with enhanced root resource acquisition. For the majority of traits in *P. nidularia f. farcata* and *B. tuldoides*, monospecific and mixed stands did not differ significantly, contrary to the hypothesis.

Leaves are the primary organs for photosynthesis and carbon capture [24]. Different bamboo stand types and silvicultural regimes significantly influence leaf functional traits [25,26]. Low leaf area and high LDMC typically reflect resource limitations on plant growth [2]. In this study, SLA and LDMC in *P. nidularia f. farcata* stands were significantly lower than in other stands, indicating constrained resource acquisition and relatively weaker adaptation to coastal sandy environments. Monospecific stands of *B. tuldoides* ‘Swolleninternode’ exhibited higher leaf thickness and lower SLA than other stands, differing from results reported for *Pinus massoniana* mixed forests [27]. This divergence likely reflects habitat differences: coastal sandy soils are nutrient-poor and subject to high temperatures and water scarcity, prompting *B. tuldoides* ‘Swolleninternode’ to adopt a more conservative strategy that allocates more photosynthate toward denser mesophyll and protective tissues, thereby reducing water loss and heat damage while improving water-use efficiency [28,29].

Higher twig wood density correlates with lower growth and water-conductance rates but greater hydraulic safety [30]. Twig TWD in monospecific stands of *B. oldhamii* significantly exceeded all other stands, indicating a “conservative” hydraulic strategy that trades conductance efficiency for safety—a meaningful adaptation to coastal wind and salt stress. TDMC in mixed stands of *P. nidularia f. farcata* exceeded monospecific stands, possibly because the mixed-stand microenvironment enhanced carbon accumulation and structural development in branches—a pattern consistent with *Betula platyphylla* × larch mixed stands [31].

Silvicultural regime also influenced root architecture [32]. Both *B. oldhamii* and *P. nidularia f. farcata* in mixed stands showed higher SRL and SRA alongside lower RTD, traits associated with enhanced resource acquisition and carbon uptake [33,34,35]. This pattern mirrors findings for *Cunninghamia lanceolata* [36] and represents an active growth strategy by which bamboo adapts to silvicultural change.

High CVs for leaf area, SLA, leaf volume, LTD, twig volume, and TWD indicate that these traits are highly sensitive to environmental stress and ecologically plastic [37,38]. Conversely, leaf thickness, LDMC, twig length, twig diameter, and TDMC showed lower CVs, reflecting stronger genetic constraints and stable environmental selection pressures that constrain within- and between-species variation [39,40]. The clonal growth habit of bamboo imposes a degree of genetic stability on functional traits, yet phenotypic plasticity remains high, enabling adaptation to heterogeneous coastal habitats [20].

### 4.2. Why Do Monospecific and Mixed Stands Show Similar Functional Traits?

Contrary to the hypothesis, most leaf, twig, and root functional traits did not differ significantly between monospecific and corresponding mixed stands of the same bamboo species (A vs. D; B vs. E; C vs. F; Figure 2 and Figure 3, Table 2). The study proposes three complementary explanations:

(1) **Genetic constraints imposed by clonal growth.** Bamboo expands clonally through underground rhizomes, which enables resource sharing (photosynthates, water, minerals) among connected ramets and imposes strong genetic regulation on functional traits [20,21]. This physiological integration may buffer individual ramets against local environmental variation, constraining trait shifts below detectable thresholds even when stand structure changes (monospecific vs. mixed).
(2) **Short establishment time and undeveloped interspecific competition.** All experimental plots had been established for ≥5 yr without silvicultural management, but this period may be insufficient for interspecific interactions between bamboo and *C. equisetifolia* to reach equilibrium. Evidence from mixed-species plantation studies shows that the facilitative or competitive effects of tree species mixing on functional traits are strongly modulated by stand age, often intensifying only after a decade or more of stand development [41,42,43]. The plots, still in early successional stages, have likely not yet accumulated sufficient interspecific interactions to drive significant trait divergence from monospecific stands.
(3) **Relatively low proportion of *C. equisetifolia* in mixed stands.** With a bamboo-to-*C. equisetifolia* mixing ratio of 7:3, the companion species contributes only 30% of stand basal area, which may be insufficient to substantially alter light, soil moisture, or nutrient availability for the bamboo component. Research on conifer–broadleaf mixed forests demonstrates that mixing ratio critically governs the direction and magnitude of interspecific interactions and tree functional responses, and low proportions of the companion species often produce negligible effects on target species traits [44]. Future studies varying the mixing ratio and extending the observation period will be necessary to identify critical thresholds for mixed-stand effects on bamboo functional traits.

While these three mechanisms (clonal integration, stand age, and mixing ratio) are consistent with the existing literature and provide a plausible framework for interpreting the results, none was directly tested in the present study. Stand age, mixing ratio, and clonal integration were not experimentally manipulated, and direct measurements of interspecific competition intensity or rhizome resource transfer were not conducted. These findings carry practical implications for shelterbelt management: over the short term, both silvicultural regimes exert similar effects on bamboo functional traits per se. Nevertheless, the long-term ecosystem-level benefits of mixed stands—including improved soil carbon and nitrogen, enhanced microbial biomass, and greater understory biodiversity—remain important justifications for mixed-stand configurations [23,45]. Moreover, the positive interaction between *C. equisetifolia* and companion species in coastal tropical zones has been shown to increase aboveground biomass and soil nutrient availability over a decade of stand development [46], suggesting that the functional trait effects this study documents here may strengthen over time. Monitoring trait dynamics over longer timescales is therefore warranted.

### 4.3. Coordinated Relationships Among Leaf, Twig, and Root Functional Traits

Bamboo plants exhibit close inter-organ functional coordination [47,48]. The strong positive correlation between twig diameter and both leaf area and leaf volume suggests that bamboo supports larger leaves by increasing twig diameter, thereby maximising photosynthetic capture and light-use efficiency under variable stand conditions [26]. The strong negative correlation between TDMC and average root diameter reflects a carbon allocation trade-off under resource limitation: reduced aboveground biomass investment is offset by thicker roots that enhance water and nutrient uptake, strengthening belowground organ function [49,50]. The strongly negative LTD–SRL and strongly positive LTD–RTD correlations reveal further leaf–root structural and functional coordination: higher LTD improves drought tolerance and mechanical strength while restricting SLA to reduce water loss; roots simultaneously increase tissue density to retain water and nutrients, while reducing SRL to concentrate root growth in resource-rich soil layers, thereby enhancing water and nutrient use efficiency [17,51,52,53,54]. Collectively, bamboo achieves holistic adaptive integration through coordinated carbohydrate allocation, structural regulation, and water–nutrient uptake strategies across leaves, twigs, and roots in coastal sandy environments.

While the PCA results are consistent with resource acquisition and conservation frameworks, this study emphasises that PCA is a descriptive, exploratory technique and does not establish causal relationships among traits. PCA identified nine key traits with absolute factor loadings > 0.75: leaf area, leaf volume, SLA, LTD, SRL, average root diameter, total root volume, total root surface area, and twig wood density (TWD). These traits collectively span leaf carbon capture, resource conservation, and root resource acquisition. Although nine key traits were identified, this number remains relatively large for differentiating stands under different silvicultural regimes, collectively spanning leaf carbon capture, resource conservation, and root resource acquisition. Monospecific stands showed higher composite scores, indicating stronger leaf–twig–root functional coordination and resource-use efficiency in the absence of interspecific competition. Future research may employ structural equation modelling (SEM) to disentangle causal relationships among traits [45], or random forest models to rank the relative contributions of individual traits to silvicultural regime responses, thereby improving the precision and interpretability of trait selection.

### 4.4. Limitations and Future Directions

This study has four main limitations. First, the modest sample size (*n* = 3 plots per stand type) may limit detection of subtle trait differences and generalizability to other coastal sandy ecosystems. Second, single-site and single-season sampling constrains assessment of spatial and temporal variation. Third, soil properties (C, N, P, moisture, salinity) were not directly measured for each stand, limiting interpretation of resource availability effects. Fourth, proposed mechanisms (clonal integration, stand age, competition intensity) were not experimentally tested.

## 5. Conclusions

Different silvicultural regimes significantly shape the functional adaptive strategies of bamboo in coastal sandy land. Among all traits examined, leaf area and twig wood density exhibited the highest phenotypic plasticity (CVs of 74.61% and 71.91%, respectively), while leaf dry matter content was the most conserved (CV = 11.29%). Two-way ANOVA revealed that silvicultural regime effects were species-dependent: mixed stands of *B. oldhamii* showed markedly enhanced root resource-acquisition capacity (higher SRL and SRA), whereas most traits did not differ significantly between monospecific and mixed stands of the same species, likely reflecting clonal genetic constraints, short stand age (≥5 years but <10 years), and low interspecific competitive intensity at the current mixing ratio (7:3).

Leaf, twig, and root functional traits were broadly correlated across organs, demonstrating coordinated, resource-conservative integration under coastal stress. PCA composite scores were consistently higher for monospecific stands, reflecting superior multi-organ functional coordination under monoculture.

For coastal shelterbelt management, these findings provide a preliminary basis for species selection and stand configuration. *B. oldhamii* is recommended for mixed-stand configurations owing to its conservative hydraulic strategy (high twig wood density) and enhanced root acquisition in mixture; monospecific *P. nidularia f. farcata* is suitable as a pioneer for sand fixation given its high composite functional trait scores. While short-term functional effects of mixing are modest, long-term ecosystem services of mixed stands—including improved soil carbon and nitrogen cycling, enhanced microbial biomass, and greater understory biodiversity—remain compelling justifications for their adoption.

Future research should investigate long-term trait dynamics across stand ages, quantify trait relationships with soil nutrient cycling and carbon storage, explore varying mixing ratios and species combinations, and employ structural equation modelling or redundancy analysis constrained by measured environmental variables to evaluate causal trait-environment relationships. Such work would provide a more robust scientific basis for precision management and sustainable restoration of coastal sandy ecosystems.

## Figures and Tables

**Table 1:** Basic stand characteristics of experimental bamboo stands.

Stand Type	Stem Height (m)	DBH (Diameter at Breast Height) (cm)	Density (100 m^2^)
Monospecific stands of *B. tuldoides* ‘Swolleninternode’ (C)	3.7 ± 0.8	1.05 ± 0.41	48.0 ± 6.0
Mixed stands of *B. tuldoides* ‘Swolleninternode’ (F)	3.1 ± 1.2	2.74 ± 0.44	77.3 ± 16.2
Monospecific stands of *B. oldhamii* (A)	3.6 ± 1.0	11.58 ± 2.66	52.0 ± 8.0
Mixed stands of *B. oldhamii* (D)	4.7 ± 1.1	4.13 ± 1.14	68.0 ± 18.3
Monospecific stands of *P. nidularia f. farcata* (B)	2.0 ± 0.6	0.37 ± 0.18	172.7 ± 11.2
Mixed stands of *P. nidularia f. farcata* (E)	2.2 ± 0.3	0.25 ± 0.06	96.3 ± 9.5

**Table 2:** Root morphological traits of monospecific and mixed bamboo stands (mean ± SD; different lowercase letters indicate significant differences, Tukey HSD, *p* < 0.05).

Stand Type	Total Root Length (m m^−2^)	Total Root Surface Area (cm^2^ m^−2^)	Average Root Diameter (mm)	Total Root Volume (cm^3^ m^−2^)	Specific Root Length SRL (m g^−1^)	Specific Root Surface Area SRA (cm^2^ g^−1^)	Root Tissue Density RTD (g cm^−3^)
Monospecific stands of *B. oldhamii* (A)	45.6 ± 22.2 b	1271.35 ± 303.84 c	1.02 ± 0.34 a	8.14 ± 3.4 b	11.2 ± 2.56 b	312.26 ± 74.63 b	0.50 ± 0.11 a
Monospecific stands of *P. nidularia f. farcata* (B)	31.15 ± 8.01 a	1570.28 ± 340.47 d	1.69 ± 0.57 c	17.27 ± 8.69 c	2.18 ± 0.52 a	109.89 ± 23.83 a	0.83 ± 0.23 b
Monospecific stands of *B. tuldoides* ‘Swolleninternode’ (C)	24.71 ± 9.24 a	923.02 ± 394.19 b	1.16 ± 0.17 ab	6.96 ± 3.42 b	6.31 ± 1.85 ab	400.99 ± 100.66 c	0.56 ± 0.15 a
Mixed stands of *B. oldhamii* (D)	27.53 ± 7.25 a	925.85 ± 309.19 b	1.05 ± 0.09 a	6.23 ± 2.53 ab	25.23 ± 5.96 c	868.7 ± 171.05 d	0.29 ± 0.09 a
Mixed stands of *P. nidularia f. farcata* (E)	20.31 ± 5.68 a	617.56 ± 214.93 a	0.96 ± 0.15 a	3.82 ± 1.75 a	3.1 ± 0.66 a	239.68 ± 32.81 ab	1.72 ± 0.47 c
Mixed stands of *B. tuldoides* ‘Swolleninternode’ (F)	22.08 ± 5.57 a	894.66 ± 286.89 b	1.28 ± 0.22 b	7.4 ± 3.29 b	3.03 ± 0.49 a	215.49 ± 39.37 ab	0.99 ± 0.36 b

Note: Data are mean ± SD. Different lowercase letters indicate significant differences among stands (Tukey HSD, *p* < 0.05).

**Table 3:** Descriptive statistics for variation in leaf and twig functional traits across bamboo stand types.

Trait	Maximum	Minimum	Median	Mean	SD	CV (%)
Leaf thickness (mm)	0.36	0.11	0.17	0.18	0.04	22.05
Leaf area (cm^2^)	96.94	5.49	17.64	24.66	18.40	74.61
SLA (cm^2^ g^−1^)	229.17	18.95	49.92	71.65	44.67	62.34
LDMC (g g^−1^)	0.39	0.24	0.31	0.31	0.04	11.29
Leaf volume (cm^3^)	1.11	0.08	0.40	0.46	0.30	64.69
LTD (g cm^−3^)	2.75	0.32	1.05	1.08	0.58	53.76
Twig length (cm)	87.40	21.20	44.45	45.60	16.19	35.50
Twig diameter (mm)	3.81	1.21	2.30	2.34	0.53	22.83
Twig volume (cm^3^)	7.54	0.29	1.80	2.14	1.40	65.24
TWD (g cm^−3^)	60.82	3.03	42.90	34.18	24.58	71.91
TDMC (g g^−1^)	0.86	0.36	0.49	0.51	0.09	18.50

**Table 4:** Principal component analysis of leaf, twig, and root functional traits.

Factor	PC 1	PC 2	PC 3	PC 4	PC 5
Leaf tissue density (LTD)	**−0.93**	−0.19	0.06	0.08	−0.15
Specific leaf area (SLA)	**0.89**	0.16	−0.31	−0.10	0.23
Leaf volume (LV)	**0.80**	0.37	−0.31	−0.17	0.28
Leaf area (LA)	**0.80**	0.32	−0.39	−0.05	0.27
Specific root length (SRL)	**0.77**	−0.14	0.10	0.57	−0.21
Root tissue density (RTD)	−0.71	−0.43	−0.33	−0.23	0.34
Specific root surface area (SRA)	0.69	−0.23	−0.22	0.39	−0.47
Twig length (BL)	−0.65	0.08	−0.25	0.58	0.28
Leaf dry matter content (LDMC)	−0.61	0.30	−0.47	0.06	0.14
Average root diameter (ARD)	−0.43	**0.82**	0.10	−0.17	−0.10
Total root volume (TRV)	−0.39	**0.81**	0.32	0.11	0.00
Total root surface area (TRSA)	−0.14	**0.79**	0.50	0.29	0.07
Twig diameter (BDia)	0.46	0.71	−0.25	−0.12	−0.07
Twig volume (BV)	−0.20	0.71	−0.48	0.26	0.01
Twig dry matter content (TDMC)	0.24	−0.71	0.09	0.21	0.26
Twig wood density (TWD)	0.30	−0.27	**0.76**	−0.18	0.32
Total root length (TRL)	0.29	0.29	0.65	0.50	0.32
Leaf thickness (LT)	0.23	0.34	0.42	−0.60	−0.17
Eigenvalue	6.206	4.390	2.631	1.794	1.015
Contribution rate (%)	34.478	24.387	14.614	9.966	5.639
Cumulative contribution rate (%)	34.478	58.865	73.479	83.445	89.084

Note: Bold values indicate absolute factor loadings > 0.75 (key traits). PC = principal component.

**Table 5:** Comprehensive evaluation of leaf–twig–root functional trait scores under different silvicultural regimes.

Stand Type	F1	F2	F3	F4	F5	F (Composite)	Rank
Monospecific stands of *P. nidularia f. farcata* (B)	−3.33	604.08	479.28	393.33	94.34	292.83	1
Monospecific stands of *B. oldhamii* (A)	1.85	468.77	367.62	382.78	8.32	232.85	2
Mixed stands of *B. tuldoides* ‘Swolleninternode’ (F)	1.07	342.79	234.15	258.14	23.46	163.13	3
Monospecific stands of *B. tuldoides* ‘Swolleninternode’ (C)	0.46	322.48	240.34	320.56	−82.19	158.67	4
Mixed stands of *B. oldhamii* (D)	2.92	281.51	156.17	466.94	−281.41	138.44	5
Mixed stands of *P. nidularia f. farcata* (E)	2.96	219.69	161.17	223.70	−32.92	108.46	6

Note: F = weighted composite score (weights = PC contribution rates). A higher score reflects greater overall leaf–twig–root functional coordination. Rank 1 = highest composite score.

## Data Availability

The data presented in this study are available on request from the corresponding author. The data are not publicly available because they form part of an ongoing long-term monitoring programme on coastal shelterbelts on Dongshan Island and are subject to institutional data-management restrictions of Fujian Agriculture and Forestry University.

## Supplementary Materials

The following supporting information can be downloaded at: https://www.mdpi.com/article/10.3390/plants15162487/s1, Table S1: Two-way ANOVA results for functional traits of bamboo stands under different silvicultural regimes.
