# Supplementary material for: Comparative Functional Traits of Bamboo Monospecific Stands and Bamboo–Casuarina Mixed Stands in Coastal Sandy Land Under Different Silvicultural Regimes
Source: Plants (Basel). 2026 Aug 16;15(16):2487. doi: 10.3390/plants15162487 (PMC13516200; doi:10.3390/plants15162487)
Supplement: Supplementary file 1 [file plants-15-02487-s001.zip › plants-4476838-supplementary.pdf]

Supplementary Material

Table S1. Two-way ANOVA results for functional traits of bamboo stands under different silvicultural regimes

Species: Phyllostachys nidularia f. farcata, Bambusa oldhamii, Bambusa tuldoides "Swolleninternode". Regime: monospecific vs. mixed with Casuarina equisetifolia (7:3 ratio). Data were log(x+1)-transformed. F-statistics, P-values, and partial eta-squared (η^2p) are reported. Values in bold indicate $P < 0.05$.

Trait	F	P	η^2p	F	P	η^2p	F	P	η^2p
		Species			Regime			Interaction	
Leaf thickness	**18.73**	**<0.001**	**0.257**	**30.45**	**<0.001**	**0.220**	0.08	0.926	0.001
Leaf area	**77.67**	**<0.001**	**0.649**	**15.36**	**<0.001**	**0.155**	**37.71**	**<0.001**	**0.473**
Specific leaf area	**57.92**	**<0.001**	**0.580**	**13.76**	**<0.001**	**0.141**	**3.46**	**0.036**	**0.076**
Leaf dry matter content	**10.56**	**<0.001**	**0.413**	2.72	0.110	0.083	**5.08**	**0.013**	**0.253**
Leaf volume	**49.16**	**<0.001**	**0.539**	**8.13**	**0.005**	**0.088**	**19.76**	**<0.001**	**0.320**
Leaf tissue density	**63.31**	**<0.001**	**0.601**	1.45	0.232	0.017	**3.77**	**0.027**	**0.082**
Twig length	**12.59**	**<0.001**	**0.375**	0.25	0.622	0.006	0.85	0.436	0.039
Twig diameter	2.31	0.112	0.099	0.49	0.489	0.011	**6.64**	**0.003**	**0.240**
Twig volume	0.88	0.422	0.040	0.01	0.930	0.000	3.20	0.051	0.132
Twig wood density	2.54	0.091	0.108	1.37	0.249	0.032	**4.82**	**0.013**	**0.187**
Twig dry matter content	**7.82**	**0.002**	**0.395**	3.20	0.086	0.118	0.84	0.444	0.065
Total root length	**32.64**	**<0.001**	**0.845**	**8.14**	**0.015**	**0.404**	**8.35**	**0.005**	**0.582**
Total root surface area	1.01	0.395	0.144	**8.60**	**0.013**	**0.417**	**22.15**	**<0.001**	**0.787**
Average root diameter	**40.18**	**<0.001**	**0.870**	**52.73**	**<0.001**	**0.815**	**92.80**	**<0.001**	**0.939**
Total root volume	**6.46**	**0.012**	**0.518**	**19.21**	**<0.001**	**0.616**	1.37	0.291	0.186
Specific root length	**10.08**	**0.003**	**0.627**	4.52	0.055	0.274	**6.33**	**0.013**	**0.513**
Specific root surface area	**60.03**	**<0.001**	**0.909**	3.43	0.089	0.222	**392.62**	**<0.001**	**0.985**
Root tissue density	**18.86**	**<0.001**	**0.759**	**5.02**	**0.045**	**0.295**	**22.40**	**<0.001**	**0.789**
